# Cerebral and extracerebral distribution of radioactivity associated with oxytocin in rabbits after intranasal administration: Comparison of TTA-121, a newly developed oxytocin formulation, with Syntocinon

**DOI:** 10.1371/journal.pone.0261451

**Published:** 2021-12-20

**Authors:** Daisuke Ishii, Michiharu Kageyama, Shin Umeda

**Affiliations:** 1 DMPK Research Department, Teijin Pharma Limited, Hino, Tokyo, Japan; 2 Business Development & Licensing Department, Teijin Pharma Limited, Chiyoda-ku, Tokyo, Japan; University of Toronto, CANADA

## Abstract

Autism spectrum disorder (ASD) is a neurodevelopmental disorder associated with deficits in social interactions/communication. Despite the large number of ASD patients, there is no drug approved to treat its core symptoms. Recently, Syntocinon (oxytocin nasal spray) has been reported to have a therapeutic effect on ASD. However, the disadvantage of Syntocinon for ASD treatment is that 6 puffs/administration are required to achieve the effective pharmacological dose. Furthermore, there are no published reports evaluating the cerebral distribution profile of oxytocin after intranasal administration. TTA-121 is a newly developed intranasal oxytocin formulation with high bioavailability produced by optimizing the physicochemical properties. In this study, we prepared the same formula as Syntocinon as the control formulation (CF), and the cerebral and extracerebral distribution of oxytocin in rabbits after single intranasal administration of ^3^H-labeled oxytocin formulations—[^3^H]TTA-121 and [^3^H]CF were examined and compared. The area under the concentration-time curve to the time of the last quantifiable concentration (AUC_t_) in the whole brain was 3.6-fold higher in the [^3^H]TTA-121 group than in the [^3^H]CF group, indicating increased delivery of radioactivity to the brain by TTA-121 than by CF. Since the distribution profiles showed no notable differences in radioactivity between the olfactory bulb and trigeminal nerve, intranasally-administered oxytocin was probably transferred to the brain via both pathways. The results also showed an increase in radioactivity in the prefrontal area and the precuneus, which are probable sites of pharmacological action as shown in clinical studies using functional magnetic resonance imaging (fMRI), confirming that intranasally-administered oxytocin could reach these tissues.

## Introduction

Autism spectrum disorder (ASD) is a neurodevelopmental disorder that affects approximately 1% of the general population [1]. Its core symptoms include deficits in social interactions and communication, in addition to restricted and repetitive behaviors [2]. Currently, there is no approved medication for the core symptoms [3].

Oxytocin is a human peptide hormone produced in the hypothalamus and released into the blood through the pituitary gland. It is associated with various physiological and neurological processes [4], and recent studies have reported that intranasally-administered oxytocin is potentially therapeutic for these symptoms, particularly those related to difficulties with social interaction and communication [5–9]. Several clinical trials have examined Syntocinon (Novartis, Switzerland) [5, 7], an oxytocin nasal spray approved to stimulate lactation in Europe. However, at least 6 puffs are required to attain the effective pharmacological dose (24 U/administration), causing dripping from the nose. This adversely affects patient compliance because its use for ASD treatment is long-term. For this reason, the development of a new intranasal formulation for easier and sufficient oxytocin delivery to the brain is an urgent and unmet need.

TTA-121 is a newly developed intranasal formulation of oxytocin prepared by optimizing the physicochemical properties, such as the osmolarity and viscosity. A Phase 1 study of TTA-121 in healthy male volunteers [10] revealed that single intranasal doses of TTA-121 from 5 to 200 U/mL (0.1 mL/spray/dose) and multiple intranasal doses of TTA-121 from 30 to 200 U/mL (0.1 mL/spray/dose × 9 days; days 1 and 9: QD, days 2–8: BID) had no safety issues and were well tolerated. Further, systemic exposure to oxytocin after single and multiple administration of TTA-121 increased in a dose-proportional manner across the 5–200 U/mL range. No accumulation of oxytocin was observed after multiple intranasal administrations. In addition, a Phase 2 study of TTA-121 in subjects with ASD is currently ongoing (NCT03466671/UMIN000031412).

The transfer of oxytocin to the brain following intranasal administration was also investigated in mice [11, 12], rats [11], monkeys [13–15], and humans [16]. The results of these studies indicated that oxytocin concentrations in the cerebrospinal fluid or dialysate of the amygdala and hippocampus with microdialysis increased after intranasal administration of oxytocin. There are, however, no published reports evaluating the distribution profile in cerebral tissues after the intranasal administration of radiolabeled oxytocin. Several clinical studies using fMRI have shown that the functional connectivity among the amygdala, prefrontal area, cingulate gyrus, and precuneus changes significantly after intranasal administration of oxytocin in subjects with ASD and healthy volunteers [17–19]. Based on these results, many researchers have considered elucidating the distribution of oxytocin to these tissues as essential for further research progress. In addition, although intranasally administered oxytocin is likely to be transferred to the brain mainly via the olfactory nerve and trigeminal nerve pathways [20, 21], to date, there is no direct evidence of the involvement of these pathways in the delivery of oxytocin to the brain. In this study, we first prepared the same formulation, qualitatively and quantitatively, as Syntocinon, as the CF. Subsequently, we compared the transfer of oxytocin to the cerebral tissues by TTA-121 and by Syntocinon and elucidated the biodistribution profiles of oxytocin and associated pathways of radioactivity delivery to the brain after the intranasal administration of [^3^H]TTA-121 and [^3^H]CF to rabbits.

## Materials and methods

### Formulation

[^3^H]Oxytocin was purchased from PerkinElmer Japan Co., Ltd. (Kanagawa, Japan). TTA-121 and CF (506.5 U/mg oxytocin each) were prepared and supplied by Teijin Pharma Limited (Tokyo, Japan). TTA-121 is a new oxytocin intranasal formulation containing a carboxyvinyl polymer with high viscosity (> 100 mPa∙s) and low osmolarity (< 200 mOsm). The CF was prepared according to the qualitative and quantitative analytical results of the commercially purchased Syntocinon. After removing the solvent of [^3^H]oxytocin using a centrifuge, TTA-121 or CF was mixed with the residue to prepare [^3^H]TTA-121 and [^3^H]CF. The contents of oxytocin in [^3^H]TTA-121 and [^3^H]CF immediately after preparation were determined with an HPLC fixed with a UV detector (LC series, Shimazu Corporation, Kyoto, Japan). The radiochemical purities of [^3^H]oxytocin in [^3^H]TTA-121 and [^3^H]CF immediately after preparation and after the completion of administration were determined with an HPLC fixed with a radioactive detector (625TR, PerkinElmer Japan Co., Ltd.). The oxytocin contents in [^3^H]TTA-121 and [^3^H]CF immediately after preparation were 41.4 U/mL and 44.0 U/mL, respectively. Radiochemical purities in [^3^H]TTA-121 and [^3^H]CF were 96.6% and 97.1% immediately after preparation and 96.7% and 97.2% after administration, respectively.

### Animals

Prior to the study, the animal use protocol was reviewed and approved by the Institutional Animal Care and Use Committee at Sekisui Medical Co., Ltd. (Ibaraki, Japan) and Teijin Pharma Limited. All animal experiments were conducted in the Drug Development Solutions Center of Sekisui Medical Co., Ltd., accredited by the American Association for Accreditation of Laboratory Animal Care (AAALAC) International. The animal experiments of the present study were performed in accordance with the Standard Operation Procedures of Sekisui Medical Co., Ltd.

Male 17-week-old Slc:JW/CSK rabbits were purchased from Japan SLC, Inc. (Shizuoka, Japan). Their body weights ranged from 3000.7 to 3115.4 g at dosing. The animals were housed individually in a stainless animal cage under controlled temperature (16–22°C), humidity (30–70%), and lighting (6:00–18:00) conditions throughout the study. The animals were given a commercially available diet for rabbits and guinea pigs (LRC4, Oriental Yeast Co., Ltd., Tokyo, Japan) once per day and public tap water freely with a water bottle.

### Experimental design

[^3^H]TTA-121 and [^3^H]CF were intranasally administered once via one puff to male rabbits at 4 U/body, which was the same dose as that for Syntocinon. The dosing formulation (100 μL) was sprayed into one nasal cavity of an animal anesthetized with intramuscular injection (0.95 mL/kg) of a mixture (14:5, v/v) of ketamine hydrochloride (Ketalar, 50 mg/mL) and xylazine hydrochloride (Selactar, 20 mg/mL) in a prone position using an intranasal administration device (pump: Classic, bottle: TIL-4ML [OC], Taisei Kako Co., Ltd., Osaka, Japan). The animal was then immediately turned to the supine position and left for approximately 15 min. The intranasal administration device was prepared by spraying five consecutive times prior to the administration.

After intranasal administration, the rabbits were euthanized by blood withdrawal from the abdominal aorta under anesthesia, and designated cerebral and extracerebral tissues (see Tables 1 and 2 for listed areas) were excised at 15, 45, and 90 min (n = 3 for each time point) to determine the radioactivity concentrations in the tissues. After a portion of the blood was centrifuged (1800 × *g*, 4°C, 15 min) to prepare plasma, 100 μL each of the blood and plasma was collected in a filter paper cup. After the other tissues were weighed and homogenized, approximately 150 mg of the homogenates was also collected in a filter paper cup. After the tissues in a filter paper cup were dried in an incubator at 40°C for 24 h or longer, the filter paper cups were combusted using an automatic combustion system (307, PerkinElmer Japan Co., Ltd.) and generated ^3^H_2_O was absorbed into a scintillation cocktail. Radioactivity in each sample was counted for 2 min using liquid scintillation counters (2500TR, 2700TR, 3100TR, 1900CA, PerkinElmer Japan Co., Ltd.).

**Table 1:** Radioactivity concentrations in tissues after single intranasal administration of [^3^H]TTA-121 to male rabbits (dose: 4 U/body).

	Radioactivity concentration (ng eq./g or mL)								
Tissue	15 min			45 min			90 min		
Prefrontal area	0.275	±	0.047	1.02	±	0.09	1.12	±	0.31
Precuneus	0.313	±	0.069	0.965	±	0.111	1.16	±	0.32
Cerebral cortex*	0.257	±	0.062	0.997	±	0.058	1.12	±	0.26
Olfactory bulb	0.353	±	0.053	1.18	±	0.12	1.13	±	0.22
Striatum	0.260	±	0.029	1.00	±	0.13	0.943	±	0.046
Hippocampus	0.236	±	0.043	0.958	±	0.049	1.03	±	0.25
Thalamus	0.267	±	0.057	0.961	±	0.106	1.06	±	0.35
Hypothalamus	0.279	±	0.097	0.781	±	0.025	0.888	±	0.182
Midbrain	0.308	±	0.086	0.944	±	0.150	1.02	±	0.22
Cerebellum	0.305	±	0.070	1.08	±	0.12	1.32	±	0.32
Medulla oblongata	0.268	±	0.054	0.825	±	0.077	0.938	±	0.192
Pons	0.236	±	0.052	0.839	±	0.107	0.878	±	0.134
Cervical spinal cord	0.178	±	0.051	0.593	±	0.047	0.664	±	0.153
Brain (others)	0.235	±	0.026	0.837	±	0.032	0.933	±	0.205
Whole brain	0.266	±	0.058	0.960	±	0.060	1.07	±	0.25
Plasma	1.09	±	0.19	1.64	±	0.18	1.29	±	0.34
Blood	0.784	±	0.188	1.26	±	0.09	0.938	±	0.220
Trigeminal nerve (part)	0.185	±	0.027	0.771	±	0.116	0.709	±	0.226
Pituitary gland	0.799	±	0.115	3.15	±	0.10	3.49	±	0.16
Esophagus	0.367	±	0.079	2.34	±	0.83	2.03	±	0.69
Trachea	1.42	±	2.06	4.95	±	4.43	1.51	±	0.96
Heart	0.550	±	0.046	1.40	±	0.18	1.45	±	0.35
Lung	1.74	±	1.79	3.16	±	1.52	3.66	±	2.65
Liver	0.469	±	0.063	2.60	±	0.58	3.10	±	0.55
Kidney	1.72	±	0.41	4.00	±	1.04	5.28	±	1.62
Adrenal gland	0.583	±	0.056	2.57	±	0.32	3.30	±	0.72
Spleen	0.719	±	0.077	2.96	±	0.32	3.25	±	0.81
Pancreas	1.35	±	0.29	13.2	±	3.2	13.5	±	6.6
Testis	0.202	±	0.021	1.08	±	0.12	1.30	±	0.36
Stomach	0.915	±	0.117	5.21	±	2.29	6.55	±	4.99
Small intestine	0.230	±	0.017	2.25	±	0.65	2.72	±	1.42
Large intestine	0.228	±	0.064	1.57	±	0.48	2.12	±	0.83

*: Except prefrontal area and precuneus

Data are expressed as the mean values ± SD of three animals.

**Table 2:** Radioactivity concentrations in tissues after single intranasal administration of [^3^H]control formulation to male rabbits (dose: 4 U/body).

	Radioactivity concentration (ng eq./g or mL)								
Tissue	15 min			45 min			90 min		
Prefrontal area	0.104	±	0.009	0.199	±	0.027	0.360	±	0.091
Precuneus	0.140	±	0.048	0.242	±	0.098	0.274	±	0.138
Cerebral cortex*	0.141	±	0.058	0.198	±	0.049	0.316	±	0.155
Olfactory bulb	0.435	±	0.029	0.472	±	0.169	1.07	±	0.93
Striatum	0.0994	±	0.0196	0.178	±	0.035	0.370	±	0.226
Hippocampus	0.113	±	0.018	0.180	±	0.032	0.298	±	0.160
Thalamus	0.112	±	0.028	0.159	±	0.035	0.302	±	0.153
Hypothalamus	0.114	±	0.023	0.169	±	0.056	0.308	±	0.079
Midbrain	0.0903	±	0.0406	0.205	±	0.082	0.281	±	0.142
Cerebellum	0.164	±	0.026	0.203	±	0.055	0.569	±	0.487
Medulla oblongata	0.0995	±	0.0123	0.166	±	0.058	0.413	±	0.299
Pons	0.111	±	0.036	0.165	±	0.067	0.286	±	0.143
Cervical spinal cord	0.0875	±	0.0306	0.135	±	0.023	0.249	±	0.148
Brain (others)	0.109	±	0.006	0.219	±	0.086	0.253	±	0.122
Whole brain	0.133	±	0.018	0.195	±	0.048	0.355	±	0.193
Plasma	0.233	±	0.043	0.384	±	0.238	0.271	±	0.107
Blood	0.175	±	0.047	0.421	±	0.286	0.376	±	0.227
Trigeminal nerve (part)	0.441	±	0.527	0.309	±	0.166	0.221	±	0.131
Pituitary gland	0.554	±	0.102	0.931	±	0.431	1.70	±	0.38
Esophagus	0.710	±	0.506	8.00	±	6.53	8.05	±	6.65
Trachea	15.4	±	13.8	28.1	±	9.4	21.3	±	18.3
Heart	0.357	±	0.216	0.660	±	0.409	0.584	±	0.301
Lung	10.2	±	2.0	20.2	±	4.6	24.8	±	27.9
Liver	0.190	±	0.028	0.636	±	0.076	1.33	±	0.70
Kidney	0.360	±	0.105	0.935	±	0.100	1.44	±	0.75
Adrenal gland	0.186	±	0.073	0.681	±	0.115	1.11	±	0.45
Spleen	0.213	±	0.092	0.691	±	0.146	1.12	±	0.48
Pancreas	0.372	±	0.112	2.76	±	0.53	5.77	±	3.59
Testis	0.0599	±	0.0164	0.224	±	0.019	0.437	±	0.209
Stomach	0.317	±	0.048	0.840	±	0.136	2.29	±	0.75
Small intestine	0.0932	±	0.0345	0.460	±	0.112	1.43	±	0.80
Large intestine	0.0904	±	0.0281	0.436	±	0.091	0.833	±	0.354

*: Except prefrontal area and precuneus

Data are expressed as the mean values ± SD of three animals.

### Data analysis

Specific radioactivity in the dosing formulation (MBq/mg) was calculated with the following equation:

$$Specific\;radioactivity=(C_{radf}\times Vol)/(C_{oxy}\times Vol/S_{act})$$

Where C_radf_ is the observed radioactivity concentration of the formulation (MBq/mL), Vol is the total volume of the dosing formulation (mL), C_oxy_ is the oxytocin contents (U/mL), and S_act_ is the specific activity of TTA-121 and Syntocinon (U/mg). The radioactivity concentration (ng eq./g or mL) was calculated from the radioactivity count in each sample based on the following equation:

$$Radioactivity\;concentration=C_{rads}/[S_{rad}/(60\times A_{sam})]$$

Where C_rads_ is the radioactivity in the assay samples (dpm), S_rad_ is the specific radioactivity in the dosing formulation (dpm/ng), and A_sam_ is the assay amount of the sample (g or mL). The radioactivity concentration in the whole brain was calculated from the radioactivity concentrations in all cerebral tissues. Pharmacokinetics (PK) parameters of maximum concentration (C_max_) and AUC_t_ were calculated from the mean radioactivity concentrations in the plasma and whole brain after the intranasal administration of [^3^H]TTA-121 and [^3^H]CF. PK analysis was conducted with Phoenix WinNonlin version 6.4 (Certara G.K., Japan).

The statistical analyses of C_max_ and AUC_t_ cannot be performed in this study. Since the tissues were collected from different animals at each time point, it is not possible to obtain the concentration-time course of individual animals. Therefore, similar to the toxicokinetic studies with small animals such as mice and rats [22, 23], C_max_ and AUC_t_ were calculated by treating the mean concentration-time course like that in an animal. Since only one numerical value can be obtained for C_max_ and AUC_t_ in each group, statistical analysis cannot be performed.

## Results

### Distribution of oxytocin-associated radioactivity after intranasal administration of [^3^H]TTA-121

Table 1 shows the radioactivity concentrations in each tissue after a single intranasal administration of [^3^H]TTA-121 to male rabbits at 4 U/body. The highest radioactivity concentrations were observed at 90 min after administration in most of the tissues. Concentrations in the olfactory bulb at 15 and 45 min and in the cerebellum at 90 min after administration were highest in cerebral tissues. Radioactivity concentrations in cerebral tissues were lower than plasma concentrations, except for cerebellum concentration at 90 min after administration. Among extracerebral tissues, radioactivity concentrations in the trachea, lung, kidney, and pancreas were higher than plasma concentrations at 15 min after administration. Radioactivity concentrations in most of the extracerebral tissues at 45 and 90 min after administration were higher than plasma concentrations, except those in the heart, testis, and large intestine at 45 min and blood and part of the trigeminal nerve at 45 and 90 min after administration.

### Distribution of oxytocin-associated radioactivity after intranasal administration of [^3^H]CF

Table 2 shows the radioactivity concentrations in each tissue after a single intranasal administration of [^3^H]CF to male rabbits at 4 U/body. The highest radioactivity concentrations were observed at 90 min after administration in most of the tissues. The radioactivity concentration in the olfactory bulb was highest in the cerebral tissues at all time points. Radioactivity concentrations in cerebral tissues were lower than the plasma concentrations at 15 and 45 min after administration, except for the olfactory bulb concentration. However, radioactivity concentrations in most cerebral tissues were higher than plasma concentrations at 90 min after administration. Among the extracerebral tissues, radioactivity concentrations in the trachea, lung, esophagus, pituitary gland, pancreas, kidney, heart, and stomach were higher than the plasma concentration. Radioactivity concentrations in all extracerebral tissues at 45 and 90 min after administration were higher than the plasma concentration, except those in the testis at 45 min and a part of the trigeminal nerve at 45 and 90 min after administration.

### PK parameters in plasma and whole brain

**Fig 1:**
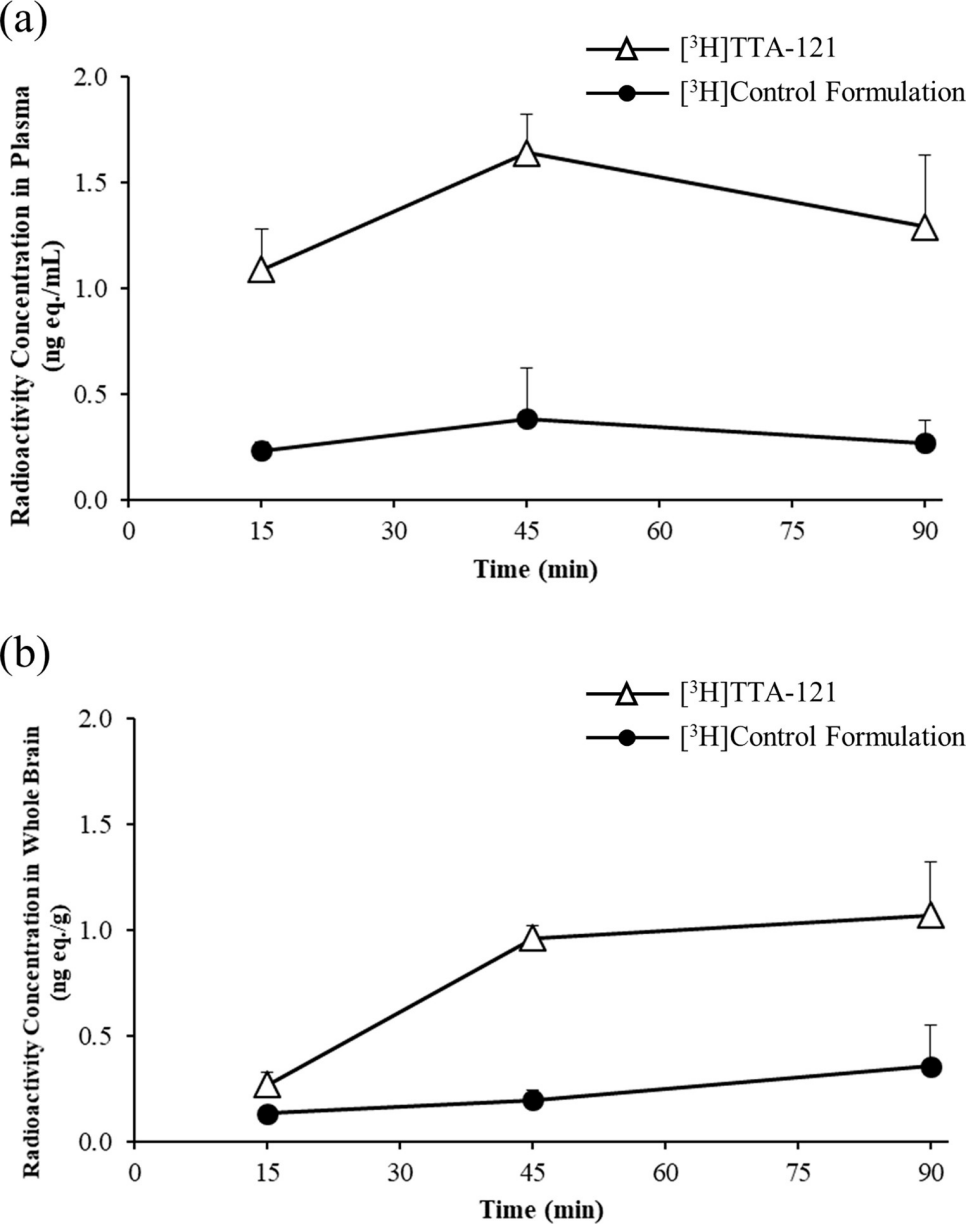
Time-course of radioactivity concentrations in (a) plasma and (b) whole brains after the intranasal administration of [^3^H]TTA-121 and [^3^H]control formulation to male rabbits (dose: 4 U/body).

Fig 1 illustrates the time-course of the mean radioactivity concentrations in the plasma and whole brain in the [^3^H]TTA-121 and [^3^H]CF groups. The mean radioactivity concentrations in the [^3^H]TTA-121 group were higher than those in the [^3^H]CF group at all time points. Table 3 shows the PK parameters in the plasma and whole brain. The C_max_ and AUC_t_ in both plasma and whole brain in the [^3^H]TTA-121 group were higher than those in the [^3^H]CF group. Compared with those in the [^3^H]CF group, the C_max_ and AUC_t_ in the [^3^H]TTA-121 group were 3.0- and 3.6-fold higher in the whole brain, and 4.3- and 4.5-fold higher in the plasma, respectively.

**Table 3:** PK parameters in plasma and whole brains after single intranasal administration of [^3^H]TTA-121 and [^3^H]control formulation to male rabbits (dose: 4 U/body).

	[^3^H]TTA-121	[^3^H]Control formulation	Ratio
C_max_ (ng eq./mL or g)			
Plasma	1.64	0.384	4.3
Whole brain	1.07	0.355	3.0
AUC_t_ (ng eq.·min/mL or g)			
Plasma	115.05	25.74	4.5
Whole brain	66.06	18.29	3.6

## Discussion

Although Syntocinon has shown efficacy for the core symptoms of ASD, particularly those related to difficulties with social interaction and communication, 6 puffs/administration are needed to deliver the effective dose. Teijin Pharma Limited has recently developed TTA-121 to improve patient compliance of intranasal oxytocin formulations. Since there has been no report on the cerebral distribution of oxytocin after intranasal administration, this study was conducted to elucidate the biodistribution profile of oxytocin after the intranasal administration of [^3^H]TTA-121 and [^3^H]CF and to compare the two formulations. This is the first report showing the cerebral distribution of radioactivity associated with oxytocin after its intranasal administration to animals.

The oxytocin contents in [^3^H]TTA-121 and [^3^H]CF immediately after preparation were around the target concentration (40 U/mL), and the radiochemical purities of [^3^H]oxytocin in [^3^H]TTA-121 and [^3^H]CF immediately after preparation and after administration were more than 95%. This result indicates that both formulations had sufficient quality during the study period. After the intranasal administration of [^3^H]TTA-121 and [^3^H]CF to rabbits at 4 U/body, the mean plasma and whole brain concentrations in the [^3^H]TTA-121 group were higher than those in the [^3^H]CF group at all time points. Further, C_max_ and AUC_t_ in the plasma and whole brain in the [^3^H]TTA-121 group were 3.0–4.5-fold higher than those in the [^3^H]CF group. Based on these results, we judged that the bioavailability of radioactivity associated with oxytocin from TTA-121 and delivery to the brain were high compared to those with Syntocinon. Considering these results, TTA-121 is expected to be effective at lower doses with a smaller number of puffs in humans. TTA-121 has a high viscosity and low osmolarity. The high viscosity facilitates increased residence time in the nasal cavity [24]. Although the mechanism has not been well studied, Vora et al. reported that the pharmacological availability of growth hormone-releasing peptide following intranasal administration with a hypotonic formulation was high compared to that with an isotonic and hypertonic formulation [25]. Therefore, it is considered that the higher TTA-121 bioavailability and transfer to the brain were attributable to these physicochemical properties.

Radioactivity concentrations in the cerebral and extracerebral tissues after the intranasal administration of [^3^H]TTA-121 and [^3^H]CF at each time point were also compared. The radioactivity concentrations in the cerebral tissues were higher in the [^3^H]TTA-121 group than in the [^3^H]CF group, except for the olfactory bulb concentration at 15 min after administration, suggesting that TTA-121 has higher transfer potential of radioactivity associated with oxytocin to the brain than Syntocinon. These results were consistent with those of the whole brain as described previously. Radioactivity concentrations in extracerebral tissues, except the esophagus, trachea, and lung at all the time points and part of the trigeminal nerve at 15 min after administration, were higher in the [^3^H]TTA-121 group than in the [^3^H]CF group. The higher concentrations in the extracerebral tissues in the [^3^H]TTA-121 group were probably attributable to high bioavailability compared to those in the [^3^H]CF group. However, the higher concentrations in the esophagus, trachea, and lung in the [^3^H]CF group were attributable to the lack of retention of radioactivity associated with oxytocin in the nasal cavity due to the lower viscosity, as this formation would have descended to these tissues after the intranasal administration of [^3^H]CF.

When focusing on the cerebral distribution profiles, we noticed that the olfactory bulb concentrations were higher than those in any other cerebral tissues in either the [^3^H]TTA-121 or [^3^H]CF group, except for the concentration in the [^3^H]TTA-121 group at 90 min after administration. However, no notable differences were observed in radioactivity concentrations among the olfactory bulb, other cerebral tissues, and part of the trigeminal nerve. Based on this result, we considered that intranasally administered oxytocin is probably transferred to the brain via both olfactory nerve and trigeminal nerve pathways. In addition, the increase in radioactivity concentrations in the prefrontal area and the precuneus indicates that intranasally administered oxytocin could reach these tissues. This finding will support the results of clinical studies using fMRI.

In conclusion, the bioavailability of oxytocin from TTA-121 and delivery to the brain were high compared to those with Syntocinon. The optimal nasal formulation design gives TTA-121 the capacity to reduce the amount of oxytocin wasted as a result of descension to the esophagus, trachea, and lung. TTA-121 is expected to be effective at lower doses with a smaller number of puffs in humans.
